# Bioelectrical activity of the pelvic floor muscles during synchronous whole-body vibration – a randomized controlled study

**DOI:** 10.1186/s12894-015-0103-9

**Published:** 2015-10-24

**Authors:** Magdalena Stania, Daria Chmielewska, Krystyna Kwaśna, Agnieszka Smykla, Jakub Taradaj, Grzegorz Juras

**Affiliations:** Department of Physiotherapy Basics, Jerzy Kukuczka Academy of Physical Education, Mikołowska 72a, 40-065 Katowice, Poland; Department of Human Motor Behavior, Jerzy Kukuczka Academy of Physical Education, Mikołowska 72a, 40-065 Katowice, Poland

**Keywords:** Electromyography, Healthy volunteers, Pelvic floor, Vibration

## Abstract

**Background:**

More and more frequently stress urinary incontinence affects young healthy women. Hence, early implementation of effective preventive strategies in nulliparous continent women is essential, including pelvic floor muscle training. An initial evaluation based on the bioelectrical activity of the pelvic floor muscles (PFM) during whole-body vibration (WBV) would help to devise the best individualized training for prevention of stress urinary incontinence in woman. We hypothesized that synchronous WBV enhances bioelectrical activity of the PFM which depends on vibration frequency and peak-to-peak vibration displacement.

**Methods:**

The sample consisted of 36 nulliparous continent women randomly allocated to three comparative groups. Group I and II subjects participated in synchronous whole-body vibrations on a vibration platform; the frequency and peak-to-peak displacement of vibration were set individually for each group. Control participants performed exercises similar to those used in the study groups but without the concurrent application of vibrations. Pelvic floor surface electromyography (sEMG) activity was recorded using a vaginal probe during three experimental trials limited to 30s, 60s and 90s. The mean amplitude and variability of the signal were normalized to the Maximal Voluntary Contraction – MVC.

**Results:**

Friedman’s two-way ANOVA revealed a statistically significant difference in the mean normalized amplitudes (%MVC) of the sEMG signal from the PFM during 60s- and 90s-trials between the group exposed to high-intensity WBV and control participants (*p* < 0.05). Longer trial duration was associated with a statistically significant decrease in the variability of sEMG signal amplitude in the study and control groups (*p* < 0.05).

**Conclusions:**

Synchronous high-intensity WBV (40 Hz, 4 mm) of long duration (60s, 90s) significantly enhances the activation of the PFM in young continent women. Prolonged maintenance of a static position significantly decreases the variability of sEMG signal amplitude independent of whole-body vibrations. Single whole-body vibrations in nulliparous continent women does not cause pelvic floor muscle fatigue.

**Trial registration:**

The trial was registered in the Australian and New Zealand Clinical Trials Registry (no. ACTRN12615000966594); registration date: 15/09/2015.

## Background

Whole-body vibration training (WBV) provides valuable assistance in sports training and physiotherapy. Exercises on a vibration platform have been used in the treatment of patients with non-specific chronic low back pain [1], Parkinson’s disease [2], multiple sclerosis [3], hemiplegia [4] and in children with cerebral palsy [5]. Some researchers also report on the use of mechanical vibration for neuromuscular stimulation of weakened pelvic floor muscles (PFM) in women with stress urinary incontinence [6, 7].

Mechanical vibration of a human skeletal muscle induces a tonic reflex contraction which is termed a tonic vibration reflex (TVR). TVR is a result of repeated, fast and short extension of a musculotendinous unit [8]. Stimulation of the endings of myelinated Ia fibres resulting from a change in the length of neuromuscular spindles causes activation of α-motoneurons in the spinal cord; consequently, the muscle contracts. Additionally, mechanical oscillations mask short-latency phasic spinal reflexes by increasing presynaptic inhibition [8].

Muscle activity is frequently analysed using surface electromyography (sEMG). EMG records and quantifies the electrical activity generated by muscle fibers. Depolarisation and repolarisation of the surface membrane of muscle fibers is the source of the electrical potential changes detected. Auchincloss et al. [9] demonstrated between-trial reliability of EMG data recorded from the PFM using two different vaginal probes with the subjects performing two tasks (Maximal Voluntary Contraction MVC and coughing). Some authors [10, 11] emphasize the need for the elimination of vibration-induced motion artifacts from raw EMG data using digital band-stop filters. Ritzmann et al. [12], on the other hand, argue that the effect of motion artifacts on EMG recording is insignificant; the periodic spikes in EMG signal during whole-body vibration are not motion artifacts, but rather stretch reflex.

Whole-body vibrations induce an increase in myoelectric activity [13, 14]. Bioelectrical muscle activity depends on several factors and among these vibration frequency and peak-to-peak vibration displacement [13], initial position in a given exercise and related initial muscle extension [14], anatomical location of the investigated muscle [15], vibration type (sinusoidal vs stochastic vibration) [6, 14] and additional load [14].

The pelvic floor muscles have typical striated fibers, specific function and characteristic location; they are characterized by synchronous and harmonic contractions [16] and prolonged tension (except for micturition and defecation) [17]. Women with stress urinary incontinence frequently exhibit weakening of the pelvic floor muscles [18]. The aim of therapeutic interventions in women with stress urinary incontinence and weakened pelvic floor muscles is to enhance muscular power so that they would be able to quickly and intensively contract these muscles to prevent involuntary urine loss in case intra-abdominal pressure should increase rapidly [6].

More and more frequently stress urinary incontinence affects young healthy women [19]. Hence, early implementation of effective preventive strategies in nulliparous continent women is essential, including pelvic floor muscle training. There is scientific basis for hypothesizing about beneficial effects of mechanical vibration on the pelvic floor muscles in continent as well as incontinent subjects [6, 7, 20]. However, such hypotheses require verification.

Previous studies had demonstrated differential effect of the sinusoidal and stochastic whole-body vibration, with the superiority of stochastic vibration especially pronounced in subjects with impaired PFMs function [6]. However, Luginbuehl et al. [7] found no significant change in the PFM activity during continuous or intermittent stochastic resonance whole-body vibration.

Literature review yields numerous reports on the effect of vibration on maximum strength and power enhancement [21]; however, the findings of these studies are not unequivocal. The discrepancies probably result from divergence in whole-body vibration training protocols. There is no evidence based on neurophysiological investigations regarding the values of vibration parameters which would produce the optimum effect in the neuromuscular system.

An increase in the activity of striated muscle during WBV [13, 14] indicates that similar effects might be expected for the pelvic floor muscles.

The aim of this study was to evaluate bioelectrical activity of the pelvic floor muscles during synchronous low- and high-intensity whole-body vibration in three trials. Another aim was to assess pelvic floor muscle fatigue during 90s whole-body vibration. The study participants were young continent women. An evaluation based on the performance and bioelectrical activity of the pelvic floor muscles during whole-body vibration would help to devise the best individualized training for prevention of stress urinary incontinence in woman. It would also facilitate the understanding of the effect of whole-body vibration on muscle performance.

## Methods

### Subjects

This was a randomized, controlled, 3 parallel-group study among thirty-six nulliparous continent women. Physical characteristics of the study participants are presented in Table 1. Young healthy adults who were not professional athletes were recruited. Exclusion criteria included a history of disequilibrium, acute inflammatory conditions and infections, epilepsy, cardiovascular diseases, acute phase of osteoarthritis, stress urinary incontinence, pregnancy, childbirth(s), pelvic surgery, diabetes, hypertension, neurological abnormalities, urinary tract infection, elevated temperature, spinal pain and Body Mass Index over 30 kg/m^2^. Candidates were presented with a comprehensive description of the aim and methods of the study. After obtaining their informed consent, a personal history was taken from each participant. Demographic data included age, height, weight, Body Mass Index, employment status. The experiment was carried out in the Department of Physiotherapy Basics and the Department of Human Motor Behavior at the Jerzy Kukuczka Academy of Physical Education in Katowice, Poland. The study was approved by the Bioethics Committee at the Academy of Physical Education in Katowice, Poland.

**Table 1:** Characteristics of the study participants

Group	Parameters of vibrations	Age [years]	Body mass [kg]	Height [cm]
		mean ± SD	mean ± SD	mean ± SD
I	2 mm/20 Hz	22.4 ± 1.6	63.5 ± 5.3	168.9 ± 2.3
II	4 mm/40 Hz	21.8 ± 1.7	64.4 ± 6.2	167 ± 3.1
III (control)	no vibrations	22.7 ± 1	63.6 ± 6.7	167.8 ± 3.7

Result distribution in a randomly selected sample was unimodal; skewness and flatness were lower than 2.5. Therefore center stratification and dispersion measure were best assessed with the arithmetic mean and standard deviation. We assumed the probability of a type I error a = 0.05, target power of 1-beta = 0.80 and a 25 % minimum significant difference between the means of parameters studied. The resultant minimum sample size was 10 patients. The target sample size was 36; 6 additional participants were to be recruited to account for dropouts (due to artifact signals of sEMG). The actual sample size was 33. The study participants were randomly assigned to 3 groups (Group I – 12; Group II – 10, and Group III – 11). Simple randomization technique was used in the experiment. The main coordinator who allocated the participants to groups had opaque, sealed envelopes, each containing a piece of paper marked with either group I, II or III. The physician selected and opened an envelope in the presence of a physiotherapist to see the symbol and then directed the participant to the corresponding group.

sEMG recordings of three women (two from group II and one from group III) were excluded from analysis due to artifact signals. Group I and II subjects participated in synchronous whole-body vibrations on a vibration platform; the frequency and peak-to-peak displacement of vibration were set individually for each group, i.e. 2 mm/20 Hz for group I and 4 mm/40 Hz for group II. In order to show a wider range of possible muscle reactions, specific combinations of low and high intensity parameters, available with the used platform, were applied. Control participants (group III) performed exercises similar to those used in the study groups but without the concurrent application of vibrations. The control participants assumed the same static position, which was standing with their knees and hips joints flexed to 35° while their arms were stretched horizontally in front to hold the railing. Hence, their position was the same as that assumed by group I and II subjects exercising on the vibration platform. Three static exercise trials of 30s, 60s and 90s were performed in a randomized order.

**Fig. 1:**
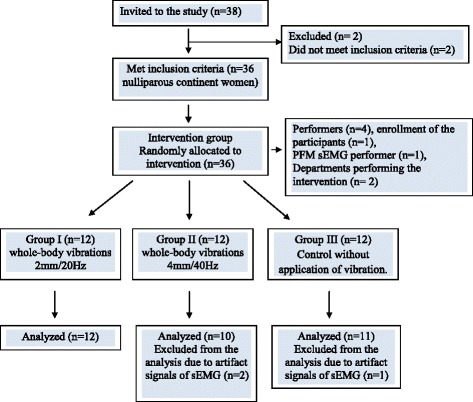
Diagram flow

The study design is presented in Fig. 1. No important difference in any characteristic was found at baseline between the groups.

### sEMG measurements

The first participant was enrolled in the study on the 6th of May 2014, the last one on the 14th of November 2014. The measurements were taken in the morning hours to minimize the impact of fatigue. The subjects were asked not to take up intensive physical exercises 24 h before the measurement. Temperature in the examination room was 24 °C. The measurements were performed under standard testing conditions, the same for all subjects.

sEMG was recorded using Myo Trace 400 (Noraxon U.S.A. Inc.) with a preamplifier (band pass filter 20 Hz–500 Hz, Common Mode Rejection Ratio of >100 dB at 60 Hz, input impedance >100 MΩ, amplifier gain 500). A 16-bit analog to digital (A/D) converter with an anti-aliasing filter set to 500 Hz frequency was also used.

Pelvic floor sEMG activity was recorded using a small diameter vaginal probe with two metal sensors (Everyway Medical Instruments Co). The probe was inserted using a small amount of antiallergic lubricant with the sensors positioned laterally in the vagina. Vaginal electrode placement was checked during breaks between the consecutive measurement sessions. After cleansing the skin site with an alcohol swab, the reference surface electrode was placed over the right anterior superior iliac spine (round self-adhesive electrode; silver/silver chloride) in accordance with SENIAM recommendations (Surface ElectroMyoGraphy for the Non- Invasive Assessment of Muscles) [22]. sEMG cables were fixed on the skin with tape in order to avoid artifacts.

Prior to the measurements, the participants were asked to urinate a full void. All sEMG recordings were performed by the same examiner.

### Whole-body vibration

Synchronous whole-body vibration was carried out on a vibration platform (Fitvibe 600, Gymna Uniphy N.V.). Test participants were in a static position during the exercises. Briefly, each subject was asked to stand on the platform, loading their feet uniformly, with the knee and hip joints bent at 35° and the upper extremities stretched horizontally forwards, holding on to a railing. The ranges of flexion of the hip and knee joints were measured with a goniometer to ensure the subjects maintained the required position. The above mentioned position is quite safe as knee flexion reduces the amount of vibration that reaches the head [10]. No unintended effects were observed during WBV in group I and II.

### Testing procedure

The experiment consisted of two phases: 1/ the maximal voluntary contractions (MVC) procedure to recruit pelvic floor muscles and 2/ three static exercise trials (30s, 60s, 90s) performed in a randomized order to determine PFM activity during (groups I, II) or without (group III) WBV.

During the first phase, each participant was instructed to perform MVC of the pelvic floor muscles as forcefully as possible for about 5 s. Three attempts were made with 60-s rests between each contraction to reduce the effect of muscle fatigue. During MVCs verbal encouragement was provided. MVCs were used as reference values.

The MVC procedure to recruit pelvic floor muscles: supine lying [23]; the hip and knee were positioned at 30° and 90° of flexion, respectively. The positions were controlled with the goniometer.

During the second phase, all group I and II participants were exposed to WBV during three exercise trials applied at random order. Vibration exposure during a single trial was limited to 30s, 60s and 90s (up to 5 s were allowed for the vibration platform to reach its preset peak-to-peak displacement and frequency). A 10-min rest period was used between trials in order to eliminate any potential fatigue; the subjects were blinded to WBV frequency and peak-to-peak displacement. sEMG signal from the pelvic floor muscles was recorded during static position maintained on a vibrating platform. During the 30-s, 60-s and 90-s sEMG recording the following parameters were measured: mean amplitude (% MVC) and variability of the signal (the variability of data around the mean value of the selected period of the analysis, expressed as %MVC).

The mean amplitude as well as the mean and median frequency of the sEMG signal [24] were additionally measured to determine the effect of fatigue during the 90-s sEMG recordings. The analysis was performed for two subperiods, ie., from the 6^th^ to 10^th^ seconds and from the 86^th^ to the 90^th^ seconds. The initial subperiod (5 s) in which the vibration platform had not reached its preset peak-to-peak displacement and frequency was excluded from analysis. Percentage changes in the mean and median frequency and mean amplitude were calculated using the formula for the difference of two subperiods. Five 1-s intervals of both subperiods yielded five values of the variables under analysis.

The raw sEMG data were full wave rectified. Root mean square values were calculated using a 100 ms sliding window. The raw sEMG signal was used for the analysis of a 90-s contraction parameters (mean and median frequency of the sEMG signal).

### Statistical analysis

The Shapiro-Wilk test was used to check the data for normal distribution, while variance homogeneity was assessed using the Levene test. Since several parameters failed to meet the assumption regarding the normal distribution of variables and variance homogeneity, nonparametric tests were used. Friedman’s two-way ANOVA for ranks was applied when the same parameter had been measured several times (k > =2) under different conditions on the same subjects. The Bonferroni post-hoc test, which reveals which means are significantly different from each other, was also performed. Inter-trial changes in the amplitude of the sEMG signal from the pelvic floor muscles were analysed using the Kruskal-Wallis ANOVA with the Tukey post hoc test.

Values are expressed as mean +/− SD. The level of statistical significance for all analyses was set at *p* < 0.05.

## Results

### Mean amplitude

**Fig. 2:**
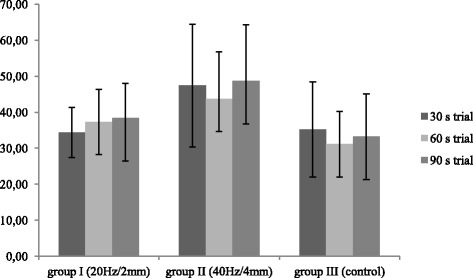
Inter-trial comparison of mean normalized amplitude of the sEMG signal from the pelvic floor muscles (%MVC) in the study and control groups

A comparison of mean normalized amplitudes between 30s, 60s and 90s trials did not reveal significant differences in any of the groups (*p* > 0.05) (Fig. 2).

Friedman’s two-way ANOVA revealed a statistically significant difference in the mean normalized amplitudes of the sEMG signal from the pelvic floor muscles during 60s- and 90s-trials between the group exposed to high-intensity WBV and control participants (*p* < 0.05) (Table 2). The mean amplitude of the sEMG signal during all trials was higher compared to the control; however, the difference did not reach statistical significance (*p* > 0.05). During all trials, the activity of the pelvic floor muscles was higher in the group who received 40Hz/4mm vibrations than in the study participants with 20Hz/2mm vibrations but again, the differences did not reach the level of statistical significance (*p* > 0.05).

**Table 2:** Comparison of mean normalized amplitude of the sEMG signal from the pelvic floor muscles (%MVC) between the groups

Mean amplitude (% MVC)							
Trial		30s trial		60s trial		90s trial	
Group	n	Mean (%)	SD	Mean (%)	SD	Mean (%)	SD
I (20Hz/2mm)	12	34.41	6.96	37.33	9.02	38.41	9.61
II (40Hz/4mm)	10	47.43	17.04	43.70	13.03	48.67	15.56
III (control)	11	35.23	13.22	31.13	9.09	33.22	11.91
p^a^(I/II/III)		0.16		0.056		0.06	
p (I/II)		0.079		0.37		0.18	
p (I/III)		0.99		0.36		0.6	
p (II/III)		0.11		0.031		0.026	

p^a^ - Friedman’s ANOVA

p - Tukey post hoc test

### Variability of amplitude

**Fig. 3:**
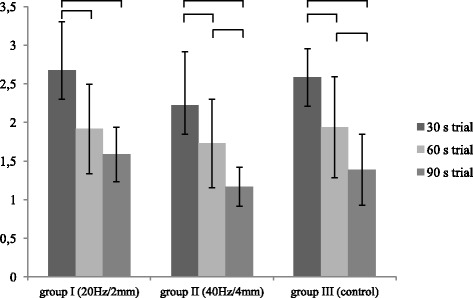
Inter-trial comparison of the normalized variability of pelvic floor muscle sEMG signal amplitude (%MVC) in the study and control groups. Horizontal bars with vertical dashes indicate statistically significant differences between duration of the trial at the same peak-to-peak displacement and frequency of vibration (*p* < 0.05)

Longer trial duration was associated with a statistically significant decrease in the variability of sEMG signal amplitude in the study and control groups (*p* < 0.05) (Fig. 3).

Kruskal-Wallis ANOVA did not reveal statistically significant differences between the groups with respect to the normalized variability of pelvic floor muscle sEMG signal amplitude (*p* > 0.05) (Table 3).

**Table 3:** Normalized variability of pelvic floor muscle sEMG signal amplitude in the study and control groups

Variability of amplitude (% MVC)							
Trial		30s trial		60s trial		90s trial	
Group	n	Mean (%)	SD	Mean (%)	SD	Mean (%)	SD
I (20Hz/2mm)	12	2.67	0.63	1.92	0.58	1.59	0.35
II (40Hz/4mm)	10	2.22	0.69	1.73	0.57	1.17	0.25
III (control)	11	2.58	0.37	1.94	0.66	1.39	0.46
p^a^(I/II/III)		0.23		0.87		0.055	
p (I/II)		0.21		0.77		0.043	
p (I/III)		0.93		0.99		0.43	
p (II/III)		0.36		0.73		0.39	

p^a^ - Friedman’s ANOVA

p - Tukey post hoc test

### Sustained 90-s contraction

In the 90s trial, absolute values (differences between the final and initial subperiods) of the median and mean frequency and mean normalized amplitude of pelvic floor muscle sEMG signal were negative, indicating a decrease in these parameters. However, intergroup differences were not statistically significant (*p* > 0.05) (Table 4). The Tukey post hoc test also did not reveal statistically significant differences.

**Table 4:** Comparison of relative changes of mean and median frequency of the sEMG signal and mean amplitude of the PFMs between the groups

		Median Frequency [Hz]		Mean Amplitude [%MVC]		Mean Frequency [Hz]	
Group	n	Mean	SD	Mean	SD	Mean	SD
I	12	−5.15	7.42	0.69	4.7	−3.47	7.2
II	11	−2.77	6.07	0.66	0.8	−4.22	5.79
III	10	−3.67	4.85	−0.18	3.18	−4.26	7.83
	p^a^(I…III)	0.86		0.45		0.99	

p^a^ - Kruskal-Wallis ANOVA

## Discussion

Surface electromyography is among the modalities to investigate the function of the pelvic floor muscles in real time [25]. sEMG was used to evaluate pelvic floor muscles’ activity in nulliparous, asymptomatic women by body position during voluntary contractions of the PFMs [26]. The reliability of repeated surface electromyography of the pelvic floor muscles was confirmed in young continent women based on the analysis of the resting, mean and peak amplitudes, time before peak and area [27].

Literature reports fail to provide consistent guidelines regarding whole-body vibration parameters. Torvinen et al. [28] emphasized the significance of appropriate frequency and amplitude selection. Using a variety of vibration protocols, the investigators demonstrated beneficial effects with respect to muscle strength and jump height after just one intervention type. The importance of appropriate parameter selection has been confirmed in the present experiment. The mean of the normalized sEMG activity of the pelvic floor muscles was significantly higher during high-intensity whole-body vibration (40 Hz, 4 mm) in the 60- and 90-sec tests compared to the control (*p* < 0.05). Low-intensity whole-body vibration did not result in significant changes in the mean sEMG amplitude of the pelvic floor muscles between the study and control groups (*p* > 0.05). Similar results were published by Lauper et al. [6]. Low-intensity whole body sinusoidal vibrations (5Hz/2mm, 5Hz/4mm,15Hz/2mm) applied in standing with slightly flexed knee joint did not enhance the activity of the pelvic floor muscles compared to standing with no vibration. A significant increase in the normalized EMG signal amplitude from the pelvic floor muscles was still observed at the combination of 15Hz/4mm.

Based on literature review, Luo et al. [21] recommend that in order to activate the muscle most effectively, vibration frequency should be in the range of 30–50 Hz. This is consistent with our results; vibrations of 40 Hz resulted in an increase in the mean activity of the pelvic floor muscles.

Increased muscle activation during whole-body vibration is mainly attributed to the tonic vibration reflex [29]. Stretching of musculotendinous units during exercise on a vibration platform causes frequency-dependent stimulation of neuromuscular spindles and, consequently, changes in the EMG signal [12]. An increase in muscle activity observed in EMG recordings results from the recruitment of a large number of motor units and high firing rates of thereof. According to Krol et al. [13], increased signal of the rmsEMG obtained while higher amplitude of vibration was applied (ie., 4 mm vs 2 mm) might be associated with faster and greater stretching of the muscle. The increase in muscle activity with increasing frequency of vibration at the same amplitude may be associated with higher rates of stretching.

Neuromuscular response to mechanical vibration also depends on the anatomical location of the muscle. Exercises performed on a vibration platform in the standing position cause an increase in bioelectrical activity, and especially in the muscles that have a distal location in the lower limb [14]. In the course of the transmission of mechanical vibrations in human tissues, the intensity of the vibration is reduced due to the damping properties which are defined as any effect that tends to decrease the amplitude of oscillation [30]. The damping effect has been attributed to joint kinematics (in particular, ankle and knee joints), muscle tuning mechanism, the sensitivity of skin receptors, viscoelastic elements and passive soft tissue and bony constraints, eg. bones, cartilage and synovial fluid [30]. Pollock et al. [31] demonstrated that vibration of 30 Hz/5.5 mm applied during standing with knee flexion (15.1°+/−4.8°) resulted in only 2.7 % of WBV acceleration being transmitted from the big toe to the head, which could be attributed to the damping effect. Above the knee at frequencies > 15 Hz, acceleration decreased with distance from the platform. It may be, that the big distance between the pelvic floor muscles and the platform as well as low vibration parameters (20 Hz, 2 mm) caused the absence of changes in the mean activity of the pelvic floor muscles during low-intensity WBV in our experiment.

Stress urinary incontinence affects women during and after menopause [32], after vaginal delivery [33], hysterectomy [34], sportswomen [35], but also - more and more frequently - young healthy women [19]. Hence, early implementation of effective preventive strategies is essential, including pelvic floor muscle training. A significantly higher activation of the pelvic floor muscles during vibration of 40 Hz and peak-to-peak displacement of 4 mm in a group of young women seems to provide an argument for the use of some elements of high-intensity vibration training in the prevention of stress urinary incontinence.

However, the knowledge on the use of vibration training in the treatment of this condition remains unsatisfactory. Sønksen et al. [20] carried out a pilot study to investigate the effect of perineal transcutaneous mechanical nerve stimulation (frequency 100 Hz and amplitude 2 mm) on the severity of stress urinary incontinence symptoms. Perineal transcutaneous nerve stimulation performed weekly for 6 weeks reduced the daily number of incontinence episodes and pad use. Seventy-three per cent of the patients reported complete resolution of stress incontinence symptoms. Lauper et al. [6] examined differences in pelvic floor muscle activation depending on the type of whole-body vibrations, ie., sinusoidal vibration and stochastic resonance vibration. The latter led to a significantly higher activation than maximum voluntary contraction, especially in post partum women with weakened pelvic floor muscles. However, in the study of Luginbuehl et al. [7] there was no significant change in PFM activity over time during stochastic resonance in women with self-reported stress urinary incontinence.

Briefly, each subject was asked to stand on the platform, loading their feet uniformly, with the knee and hip joints bent at 35° and the upper extremities stretched horizontally forwards, holding on to a railing.

With an increase in trial duration, both the study and control groups exhibited a significant decrease in the variability of sEMG signal amplitude from the pelvic floor muscles (*p* < 0.05). Body alignment for static exercise, with the knees flexed to 35° and upper limbs stretched out forwards, is a rarely assumed, unnatural and uncomfortable position. Balance control during body sway from the vertical position requires precise neuromuscular coordination and the involvement of various structures of the central and peripheral nervous systems [36]. It may be that a 30 s trial was not enough for the controller (nervous system) and responder (locomotor system) to coordinate and stabilize their actions in a nontypical body position. Since our findings revealed a decrease in the variability of sEMG signal amplitude with longer trials, we believe that an analysis of bioelectrical activity of muscles during whole-body vibration should be based on longer vibration times.

In order to assess measurement variability, Grape et al. [27] calculated the standard error of measurement. The variability between two sEMG sessions carried out on the same day with 30 min apart was smaller compared to the third performer 26–30 days later. However, due to the lack of normalization, a comparison between Grape et al.’s and our results is not possible.

EMG-fatigue slopes provide a non-invasive and standardized method to estimate neuromuscular fatigability of skeletal muscles. Mean and median frequency and the amplitude of EMG signal are among the parameters of the complete power range used in the assessment of muscle fatigue [24]. A 450-s contraction of the biceps brachii at 25 % MVC resulted in a decrease of median and mean frequency and an increase in mean amplitude thus confirming the development of muscle fatigue [24]. In our young and continent participants, 90 s vibration did not cause pelvic floor muscle fatigue. A single 90 s vibration session in young healthy women was probably not sufficient to cause pelvic floor muscle fatigue. Nevertheless, the question requires further investigations.

An analysis of raw pelvic floor EMG data revealed significantly lower values of absolute mean amplitude and mean and median frequency in women with weakened pelvic floor muscles compared to healthy participants [6]. The authors believe that such results are due to decreased muscle fiber recruitment and synchronization and low muscle strength and power in postpartum women. Luginbuehl et al. [7] demonstrated no significant change in the value of amplitude and median frequency of the EMG during stochastic resonance whole body vibration. According to the authors, it can be due to a no more than moderate to submaximal PFM activity during stochastic resonance whole body vibration.

Pelvic floor muscle fatigue has been investigated by several other authors [19, 37–40]. It was also evaluated based on changes in vaginal pressure during MVC measurement [19], perineal ultrasound to assess bladder neck motility [39], time-to-fatigue test with a relative force of 80 % [40] and Borg Scale of Perceived Exertion [38].

The neuromuscular systems alters the recruitment strategies and motor unit firing frequencies during prolonged fatiguing contractions [41]. A single 90 s vibration session in young healthy women was probably not sufficient to cause pelvic floor muscle fatigue in this experiment.

Our study has several limitations including a relatively small number of study participants. Also, we could not compare the results with incontinent women as no comparison group had been formed. Finally, we did not measure the actually generated vibration parameters and skidding of the feet as recommended by the International Society of Musculoskeletal and Neuronal Interactions [42].

## Conclusions

Our findings seem to indicate that high-intensity whole-body vibrations (frequency 40Hz, peak-to-peak displacement 4 mm) of long duration (60s, 90s) increase the mean amplitude of sEMG signal from the pelvic floor muscles in young continent women. Single 90s whole-body vibrations in young healthy women does not cause pelvic floor muscle fatigue.

The findings of the present study may have implications for clinical practice and for public health policy in terms of preventive strategies of stress incontinence in women. A significant decrease in sEMG amplitude variability suggests that research should involve long exposure to whole-body vibrations.

However, we would like to emphasize the need for further multidirectional studies on changes in pelvic floor muscle activation during whole-body vibration in women with a history of stress urinary incontinence.

## Abbreviations

PFM: Pelvic floor muscles
WBV: Whole-body vibration
sEMG: Surface electromyography
MVC: Maximal Voluntary Contraction
